# Associations of Maternal and Paternal Parenting Practices With Children’s Fruit and Vegetable Intake and Physical Activity: Preliminary Findings From an Ecological Momentary Study

**DOI:** 10.2196/38326

**Published:** 2022-08-10

**Authors:** Nanette Virginia Lopez, Mark HC Lai, Chih-Hsiang Yang, Genevieve Fridlund Dunton, Britni Ryan Belcher

**Affiliations:** 1 Department of Health Sciences Northern Arizona University Flagstaff, AZ United States; 2 Department of Psychology University of Southern California Los Angeles, CA United States; 3 Arnold School of Public Health University of South Carolina Columbia, SC United States; 4 Department of Population and Public Health Sciences Keck School of Medicine University of Southern California Los Angeles, CA United States

**Keywords:** parenting, ecological momentary assessment, fruit and vegetable consumption, physical activity, pediatrics, obesity

## Abstract

**Background:**

Childhood obesity prevention interventions routinely focus on changing maternal parenting practices. Failure to assess how fathers’ weight-related (ie, diet and physical activity) parenting practices contribute to children’s energy balance behaviors limits the understanding of their paternal role within the family. Examining the independent and interacting effects of fathers’ and mothers’ weight-related parenting practices on children’s diet and physical activity addresses this important research gap.

**Objective:**

This study used ecological momentary assessment (EMA) to investigate the within-subject and between-subject independent and interactive effects of maternal and paternal encouragement to eat and preparation of fruits and vegetables (F/V) and encouragement of and taking their child to be physically active on their child’s self-reported F/V intake and physical activity engagement.

**Methods:**

Participants included mother-father-child triads (n=22 triads, n=205-213 prompts/occasions) in the Mothers and Their Children’s Health Study and the University of Southern California Fathers Study. Simultaneously, mothers and fathers (ages_mean_ 44.2 years, SD 5.6, and 45.2 years, SD 8.1, respectively), and their children (age_mean_ 12.0 years, SD 0.7) completed up to 8 randomly prompted EMA surveys per day on separate smartphones for 7 days. At each prompt, mothers and fathers each reported whether they did the following in the past 2 hours: (1) encouraged their child to eat F/V, (2) prepared F/V for their child, (3) encouraged their child to be physically active, or (4) took their child to be physically active. Children self-reported whether they consumed F/V or were physically active in the past 2 hours.

**Results:**

Results from Bayesian multilevel logistic models (all in log-odd units) indicated that at the within-subject level, greater maternal encouragement (*β*=2.28, 95% CI 0.08 to 5.68) of eating F/V was associated with greater child report of eating F/V, but paternal encouragement (*β*=1.50, 95% CI –0.83 to 4.52) showed no effects above and beyond maternal encouragement. Additionally, greater than usual paternal encouragement (*β*=2.28, 95% CI 0.08 to 5.54) and maternal encouragement (*β*=2.94, 95% CI 0.36 to 6.69) of physical activity had significant independent effects and were associated with greater child report of physical activity. No other within-subject or between-subject associations nor interactive effects were significant.

**Conclusions:**

Findings from this study suggest that fathers play a role in supporting their children’s physical activity but not their intake of F/V. Future EMA studies should recruit larger samples to evaluate the independent and interacting roles of mothers’ and fathers’ weight-related parenting practices on child’s obesogenic behaviors.

## Introduction

Child obesity prevalence has increased over the past five decades, with 41% of US children classified as overweight, obese, or severely obese [1]. Adverse health outcomes among obese children include hypertension and hypercholesterolemia [2], which track from childhood into adulthood, placing these children at greater risk of early mortality. Parents play a critical role in shaping their children’s weight-related behaviors [3]. Thus, childhood obesity prevention interventions have focused on parental behavior change. However, these interventions have demonstrated limited success with preventing increases in children’s BMI [4]. One reason may be that the vast majority of these interventions address the practices and behaviors of mothers [5] while excluding the influential role of fathers within the family.

Research informed by family systems theory indicates that behaviors among family members are part of an interrelated system that cannot be examined in isolation [6]. Studies have explored the key role mothers play in providing support for children’s healthy eating and physical activity. Cross-sectional between-subjects research examining the relationships between the home environment and children’s fruit and vegetable (F/V) consumption showed that parental encouragement of F/V consumption was positively associated with children’s F/V consumption [7-10]. Between-subjects results from an ecological momentary study showed that children whose mothers prepared more F/V compared to other mothers had greater odds of eating F/V. Within-subjects results from this study indicated that when mothers expressed greater encouragement and preparation of F/V than usual, their children had greater odds of eating fruits and vegetables at the next prompt [11]. Using the same participants from the aforementioned study, children whose mothers reported taking their children someplace to be physically active engaged in more moderate-to-vigorous physical activity (MVPA). When mothers reported taking their children someplace to be physically active more than usual, their children engaged in more MVPA [12]. These results highlight the influential role mothers have in energy balance behaviors of their children.

Due to limited research conducted among fathers, missing from these analyses are the effects of paternal parenting practices on children’s energy balance behaviors [5,13]. This gap is concerning because fathers are parenting and caring more for their children, with a reported increase from 2.5 to 7.3 hours per week over the last 45 years [14], perhaps due to the increased percentage of working mothers, up from 47% in 1975 to 70% in 2014. Furthermore, 2 million US fathers are stay-at-home dads, up from 1.1 million in 1989 [15]. Although the number of stay-at-home dads in the United States increased 100% in the past 21 years, extant research regarding the role of fathers in children’s obesity risk is limited, due in part to the difficulty in recruiting fathers for child health studies [16].

These concerns are further exemplified by a recent review examining fathers’ role in children’s physical activity that indicated only 1.5% of observational between-subject studies conducted between 2009 to 2015 met study criteria that included the following: included fathers as study participants, presented fathers’ data separately from mothers’ data, and collected data on fathers’ physical activity parenting behaviors and/or fathers’ physical activity and children’s physical activity. The authors concluded that among the associations examined, more than half were positive, albeit modest, associations between fathers and their children’s physical activity [17]. For example, one article reviewed indicated that explicit modeling of physical activity by fathers was positively associated with their sons’ accelerometer-measured MVPA and vigorous physical activity [18]. A systematic review of 23 between-subject studies examining fathers’ feeding behaviors concluded that paternal modeling of healthy eating, own energy intake, and limit setting of unhealthy eating had a positive effect on children’s diet and reinforcement for healthy choices provided by the mother and father were positively associated with children’s healthy dietary choices [19]. This emerging evidence warrants further examination to determine how fathers’ own diet and physical activity along with their dietary and physical activity parenting practices influence children’s diet and physical activity.

Parenting practices may vary within days and across days due to interpersonal interactions, situational encounters, and changing demands and expectations and may negatively affect children’s obesogenic behaviors [20,21]. Research is beginning to explore how daily variability in parenting practices contributes to children’s obesity risk [22,23]. Participation of fathers in child obesity prevention studies poses challenges including the lack of targeted recruitment specific to fathers, time commitments that overlap with employment schedules, and failure to focus on long-term benefits of fathers’ participation [24]. To the best of our knowledge, no studies have examined the effects of within-day variability in fathers’ parenting practices on children’s energy balance behaviors (ie, diet, physical activity), resulting in a significant gap in childhood obesity research. Failure to assess how fathers’ weight-related parenting practices contribute to children’s energy balance behaviors limits the understanding of their parental role within the family.

In addition to the lack of research on fathers’ influence on their children’s energy balance behaviors, prior studies are also limited because they only assess between-subject effects, which limits our understanding of when parents may have greater influence on children’s energy balance behaviors and precludes the tailoring of intervention strategies. Ecological momentary assessment (EMA) is a methodology that uses an intense longitudinal design to collect self-reported data multiple times per day over multiple days. A prompting schedule for participants to respond to mobile phone–based surveys over the course of the day was developed specifically for the individual study. Using EMA allows for real-time data capture of parenting behaviors that increases ecological validity and addresses the lack of examination of within-subject variability. It can capture within-day fluctuations for constructs that change frequently throughout the day (eg, stress). EMA allows for disentangling of within-subjects and between-subjects effects that may ultimately assist in designing effective parenting interventions that can adjust for intra-individual differences. Additionally, EMA helps reduce recall bias found in retrospective studies as reliance on memory to inform on performed behaviors is reduced through the frequent prompting schedule. This exploratory study used EMA to investigate the within-subject and between-subject independent and interactive effects of maternal and paternal encouragement and preparation of F/V and encouragement of and taking their child to be physically active on their child’s self-reported F/V intake and physical activity engagement. Based upon cross-sectional studies that examined the role of mothers and fathers on children’s physical activity, we hypothesize that there will be a significant positive relationship between fathers’ parenting behaviors and their children’s F/V intake and physical activity independent of mothers’ parenting behaviors. Further, we hypothesize that there will be a significant relationship between mothers’ parenting behaviors and their children’s F/V intake and physical activity. We hypothesized that there would be a significant interactive effect of maternal and paternal behaviors on children’s F/V intake and physical activity.

## Methods

### Study Design and Participant Characteristics

Participants included a subsample of mother-child dyads and fathers (n=22 mother-father-child triads) enrolled in the Mothers and Their Children’s Health Study (MATCH; mother-child dyads) who were also enrolled in the University of Southern California (USC) Fathers Substudy. Participants enrolled in the MATCH study and Fathers Substudy lived in the greater Los Angeles area. Inclusion criteria for the MATCH study comprised mother having at least 50% custody, children aged 8 to 12 years, and the mother and child having the ability to read and write in English or Spanish. Exclusion criteria for the MATCH study included mother currently pregnant, mother works more than 2 weekday evenings or works on weekend days, mother or child taking medications for a psychological condition or oral or inhalant corticosteroids, mother or child experiencing health issues that prevent or limit physical activity, and child enrolled in a special education program. Inclusion criteria for the Fathers Substudy comprised father/father figure with a child currently participating in the MATCH study, having at least 50% custody, and the ability to read and write in English or Spanish. The objective of the larger MATCH study was to examine the long-term effects of mothers’ stress on their children’s energy balance behaviors (ie, diet, physical activity, sedentary time). The goal of the USC Fathers Substudy was to examine the role of fathers’ weight-related parenting behaviors on their children’s energy balance behaviors. MATCH received approval through the USC institutional review board and Northeastern University; the USC Fathers Substudy received approval through the USC institutional review board.

Mothers and their children participated in MATCH over a 3-year period, with 7-day data collection waves occurring every 6 months [25]. During the final 7-day data collection wave of the MATCH study in spring 2018, fathers were contacted via email and phone to determine interest in participating in the USC Fathers Substudy. If fathers expressed interest in participation, they were asked to accompany their spouse/child’s mother and child to their upcoming scheduled MATCH data collection appointment at which fathers reviewed and completed informed consent with research staff. Fathers without Android phones were provided moto g (Motorola Mobility LLC) smartphones for collecting EMA data. If participants owned an Android phone, they were instructed to download the appropriate app. Effects of siblings and peers of the enrolled child in the MATCH study were not included in this study.

Mothers, fathers, and their children completed up to 8 randomly prompted EMA surveys per day on their respective smartphones for 7 days. Weekday prompts began between 3 PM to 4 PM, ending between 7 PM to 8 PM for the child (total of 3 prompts) and 9 PM to 10 PM for the mother and father (total of 4 prompts). No prompting occurred during school hours. Weekend prompts started between 7 AM to 8 AM, ending at comparable times for weekday prompts. At each prompt, mothers and fathers independently reported whether they spent time with their child in the past 2 hours by answering the question, “Over the last 2 hours, have you spent time with your child (together in the same location)?” If a yes response was indicated, mothers and fathers subsequently independently reported if they did the following in the past 2 hours: (1) encouraged their child to eat F/V, (2) cooked or prepared F/V for their child, (3) encouraged their child to be physically active, and (4) took their child to be physically active. Response options for each of the 4 questions were yes or no. At each prompt, children reported if they did the following in the past 2 hours: (1) consumed F/V and (2) participated in exercise, sports, or physical activity. Response options for each of the 2 questions were yes or no. In addition to the EMA surveys, fathers completed paper surveys that included sociodemographic information (eg, age, marital status). Mothers previously completed paper surveys that included sociodemographic information (eg, age, marital status, child sex, and ethnicity).

### Ethics Approval

MATCH received approval through the USC institutional review board and Northeastern University (HS-12-00446); the USC Fathers Substudy received approval through the USC institutional review board (HS-17-00797). No other approvals were necessary for study completion.

### Statistical Analyses

Participant sociodemographics were analyzed using SPSS (version 27, IBM Corp). Means and standard deviations were calculated for mothers, fathers, and children’s ages. Percentages were determined for parents’ marital status and child sex and ethnicity.

For each child outcome (ie, whether children consumed F/V or were physically active; 1=yes, 0=no), we separately examined whether parental encouragement for eating F/V and physical activity and parental support (ie, cooking or preparing F/V and taking the child to be physically active) had predictive power. The data had a nesting structure with repeated EMA observations (level 1) nested within families (level 2). Given the small number of clusters (ie, families in our data) and the focus on binary outcome variables in our study, we used Bayesian estimation with weakly informative prior distributions to improve the stability of the results [26]. For each child outcome, we fitted 2 two-level Bayesian multilevel logistic models with repeated EMA observations nested within families and the following structure:



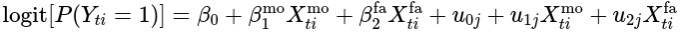



where *t* indicates time and *i* indicates family, and *X^mo^* and *X^fa^* are binary predictors for mother and father encouragement or support, respectively. The *β*s are the fixed effects and the *u*s are the random effects. To account for the potential time dependence, we also specified an autoregressive error [1] structure for observations within the same day.

For each outcome, we entered the predictors in 3 steps. In step 1, we included the intercept and the main effects of person-mean centered maternal (*X^mo^*) and paternal (*X^fa^*) encouragement/support at the occasion level as well as mean levels of maternal and paternal encouragement/support for each family. The use of family-mean centering allows us to decouple the between-family and within-family associations [27]. In step 2, we tested the interaction between the father and the mother variables.

For each model, we analyzed only observations where the child answered the EMA prompt and both parents also answered the prompts within the same 2-hour period. Observations were excluded if only one of the parents answered the prompt.

We used the R package (R Foundation for Statistical Computing) brms [28] to perform Markov chain Monte Carlo sampling. For each model, we used 4 chains, each with 2000 iterations. The first half of the samples were used as warmups, resulting in 4000 total Markov chain Monte Carlo samples. All models achieved convergence, as indicated by the potential scale reduction factor being less than 1.01 [29]. Posterior means were used as point estimates, and coefficients were considered significant statistically when the 95% credible interval excluded zero.

## Results

The total number of prompts sent was 765 for fathers, 692 for mothers, and 561 for children. The response rates were 80.0% (612/765) for fathers, 85.0% (588/692) for mothers, and 85.9% (482/561) for the child. The number of prompts where all 3 members in a family answered in the same 2-hour interval was 322, but there were missing data for specific items as reflected below in the model-specific sample sizes.

Mothers and fathers were comparable in age (ages_mean_ 44.2, SD 5.6, and 45.2, SD 8.1, respectively). Children had a mean age of 12.0 (SD 0.7) years. A total of 73% (16/22) of parents indicated being married or living as married. Of the 22 children, 55% (12/22) were female and 41% (9/22) identified as Hispanic/Latino. Child BMI z-scores had a mean value of 0.17 and ranged from –2.5 to 2.0. Table 1 shows the correlations among the parental variables and the child outcomes. We found strong correlations between encouragement and support from the same parent for the same child outcome (*r*=.55 to *r*=.83). In addition, a parent’s encouragement/support for physical activity was moderately correlated with the same parent’s encouragement/support for eating F/V (*r*=.09 to *r*=.53). There were also moderate correlations between father’s and mother’s encouragement/support (*r*=.13 to *r*=.38). Child reports of being physically active and eating F/V were moderately associated with parental encouragement and support (*r*=.09 to *r*=.26).

**Figure 1:**
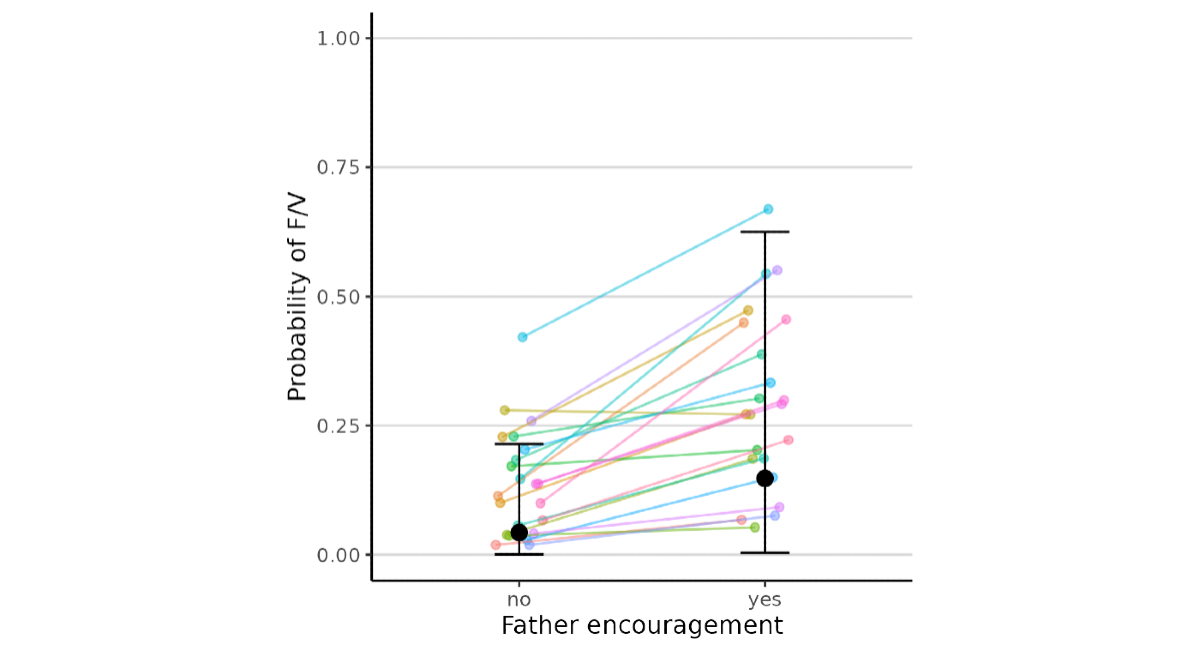
Model-predicted probabilities of a child eating fruits and vegetables as a function of father encouragement when there was no mother encouragement. The black dots and error bars show the overall predicted probabilities and the 95% confidence intervals, whereas the lines show the family-specific predicted probabilities in our sample. F/V: fruits and vegetables.

**Figure 2:**
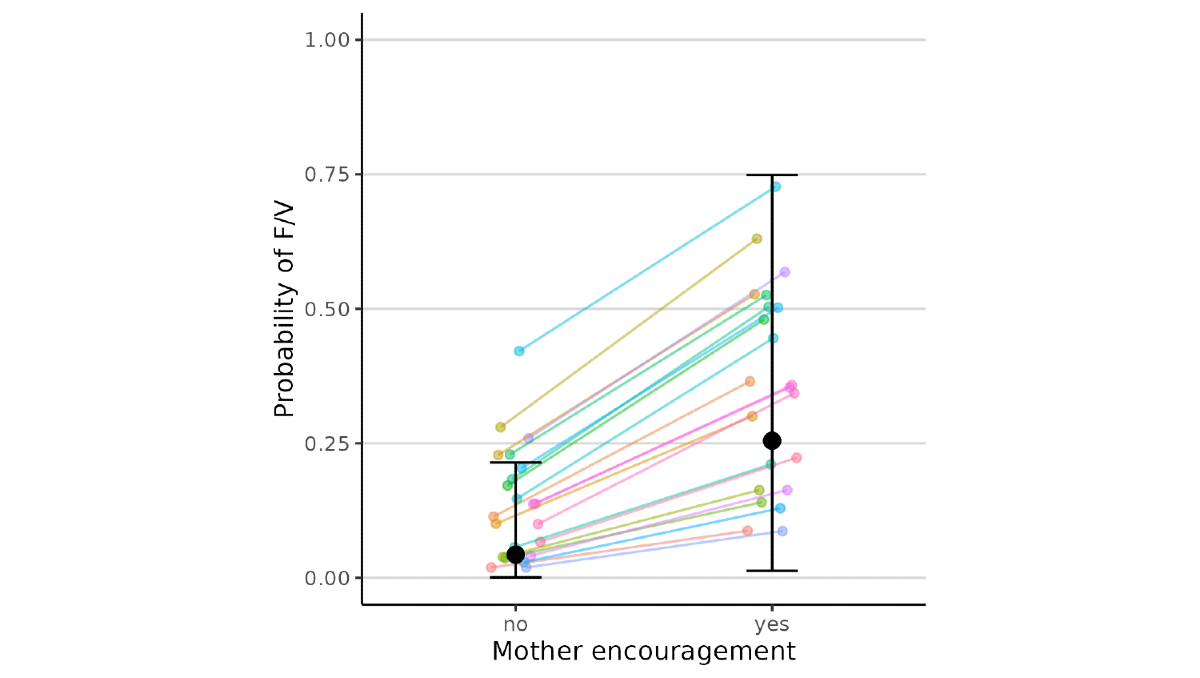
Model-predicted probabilities of a child eating fruits and vegetables as a function of mother encouragement when there was no father encouragement. The black dots and error bars show the overall predicted probabilities and the 95% confidence intervals, whereas the lines show the family-specific predicted probabilities in our sample. F/V: fruits and vegetable.

As shown in Table 2, results from Bayesian multilevel logistic models indicated that, at the occasion level, greater than usual maternal encouragement (*β*=2.28, 95% CI 0.08 to 5.68, in log-odds units) for eating F/V was significantly associated with greater likelihood of child report of eating F/V, but evidence was not significant for paternal encouragement (*β*=1.50, 95% CI –0.83 to 4.52) above and beyond maternal encouragement. Figures 1 and 2 show the model-predicted overall and family-specific probabilities with only mother’s or father’s encouragement reported in the brms package, computed using the model coefficients (ie, probability = 1 / [1 + exp(–η)] where η is the model predicted log-odds). Based on the model, when there was no father encouragement, the predicted probability of eating F/V was 0.39 with mother encouragement, compared to 0.22 without mother encouragement. However, the coefficient for paternal encouragement was not significant, and thus, only predicted probability of eating F/V with mother encouragement is reported. No statistical evidence was found for the between-family level associations, and the interactions between maternal and paternal encouragement for F/V intake were not significant.

**Figure 3:**
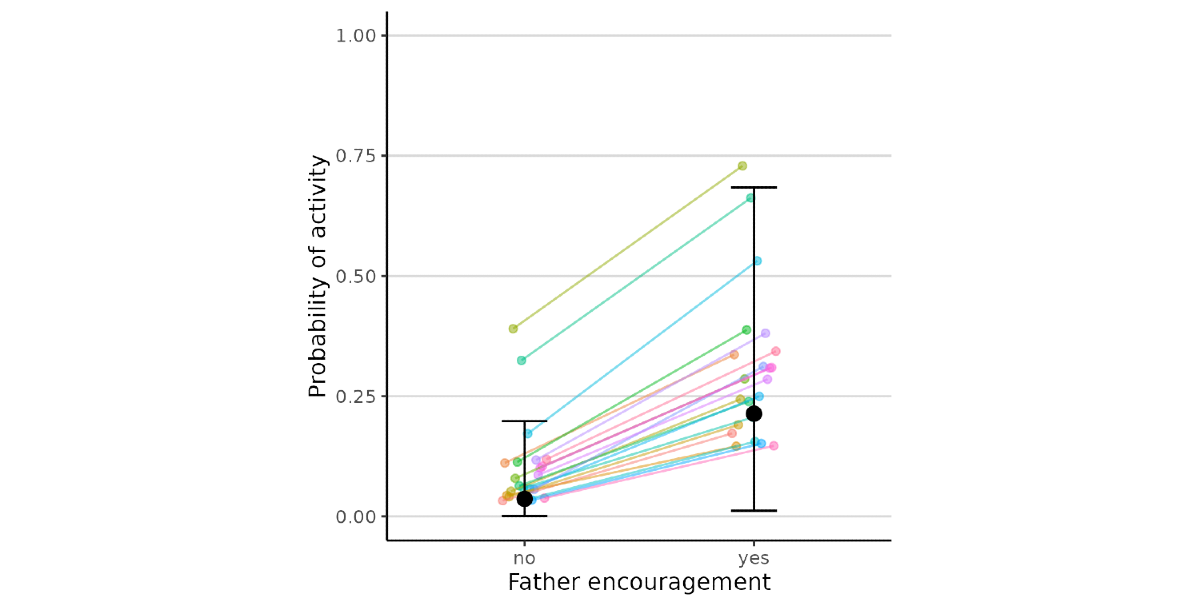
Model-predicted probabilities of a child engaging in physical activity as a function of father encouragement when there was no mother encouragement. The black dots and error bars show the overall predicted probabilities and the 95% confidence intervals, whereas the lines show the family-specific predicted probabilities in our sample.

**Figure 4:**
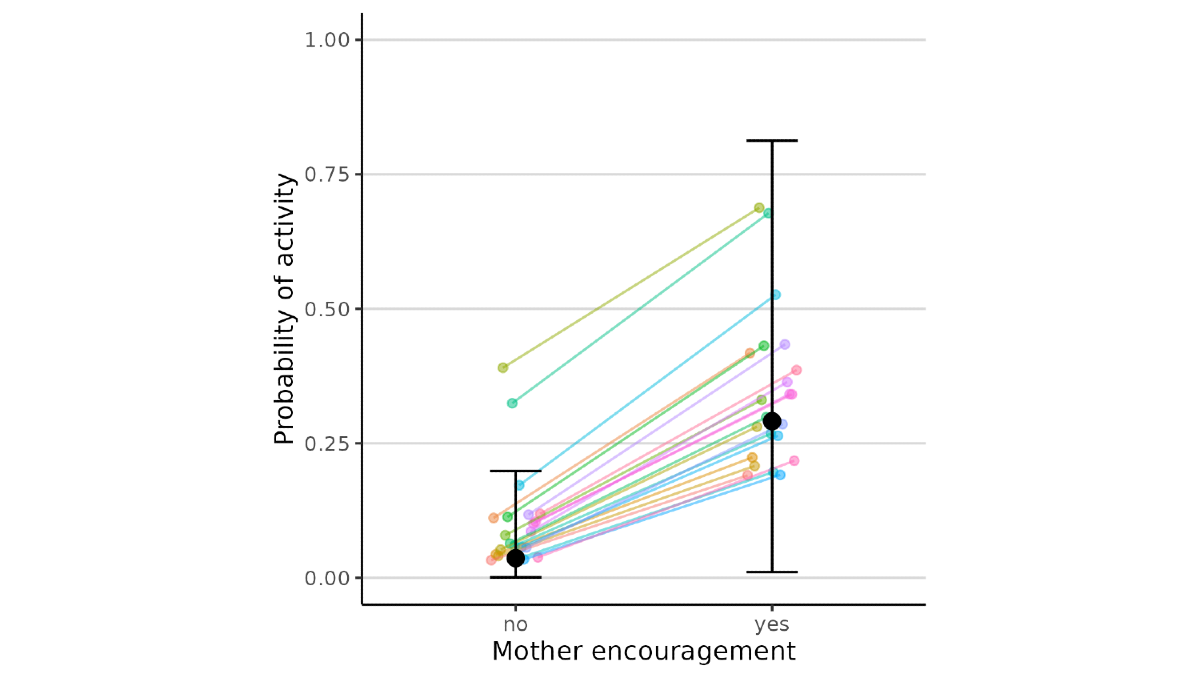
Model-predicted probabilities of a child engaging in physical activity as a function of mother encouragement when there was no father encouragement. The black dots and error bars show the overall predicted probabilities and the 95% confidence intervals, whereas the lines show the family-specific predicted probabilities in our sample.

When fathers (*β*=2.28, 95% CI 0.08 to 5.54) and mothers (*β*=2.94, 95% CI 0.36 to 6.69) each have higher levels of encouragement of physical activity than their usual level, children were more likely to report physical activity in the past 2 hours. In other words, holding mothers’ encouragement as constant, fathers’ encouragement was positively associated with children’s physical activity (individual responses shown in Figures 3 and 4 ). There were also significant differences in the coefficients of maternal and paternal encouragement across families, as indicated by the significant random effect standard deviation (for father, estimate=1.14, 95% CI 0.04 to 3.61; for mother, estimate=2.40, 95% CI 0.15 to 6.62). Specifically, the predicted probability of the child performing physical activity was 0.21 with neither mother nor father encouragement, 0.42 with only mother encouragement, 0.37 with only father encouragement, and 0.59 with both father and mother encouragement. No statistical evidence was found for differential associations at the between-subject and the within-subject levels, and the interaction between maternal and paternal encouragement for physical activity was not significant. The nonsignificant between-level coefficients indicate insufficient evidence for different between-level and within-level coefficients. The random effects were similar in Stage 1 models and were only reported in those final models (Table 2).

F/V: fruits and vegetables.

Table 3 shows the model results when using parental support (preparing F/V for the child and taking the child to be physically active) as predictors of children’s reported health behaviors. Although some of the coefficients were in the positive direction, none of them were significant. The nonsignificant between-level coefficients indicate insufficient evidence for different between-level and within-level coefficients. Parental support refers to mother/father preparing fruits and vegetables or taking the child somewhere to be physically active. The random effects were similar in stage 1 models and were only reported in those final models (Table 3).

**Table 1:** Pearson correlations among parental encouragement and support and child outcome variables.

	1	2	3	4	5	6	7	8	9
Father encouraging child to play	—^a^	—	—	—	—	—	—	—	—
Father taking the child to play	.67	—	—	—	—	—	—	—	—
Father encouraging child to eat F/V^b^	.20	.15	—	—	—	—	—	—	—
Father preparing F/V	.18	.09	.55	—	—	—	—	—	—
Mother encouraging child to play	.28	.36	–.02	–.14	—	—	—	—	—
Mother taking the child to play	.25	.38	–.03	–.13	.83	—	—	—	—
Mother encouraging child to eat F/V	.14	.14	.22	.13	.53	.44	—	—	—
Mother preparing F/V	–.01	.09	.22	.13	.25	.29	.75	—	—
Child physically active	.12	.19	–.02	–.04	.26	.24	.03	–.03	—
Child F/V consumption	.07	.13	.17	.09	.11	.13	.26	.18	.25

^a^Not applicable.

^b^F/V: fruits and vegetables.

**Table 2:** Multilevel model results (in log-odds) of parental encouragement for fruit and vegetable consumption and encouragement to be physically active predicting child outcomes of fruit and vegetable consumption and physical activity (n=205).

			Child F/V^a^ consumption	Child physical activity
**Fixed effects**				
	**Step 1**			
		Intercept	–2.88 (–6.63 to –0.01)	–2.31 (–5.62 to –0.05)
		Father encouragement (within)	1.50 (–0.83 to 4.52)	2.28 (0.08 to 5.54)
		Mother encouragement (within)	2.28 (0.08 to 5.68)	2.94 (0.36 to 6.69)
		Father encouragement (between)	0.81 (–4.61 to 7.03)	–0.49 (–5.92 to 4.64)
		Mother encouragement (between)	0.94 (–4.01 to 6.56)	–0.49 (–5.92 to 4.64)
	**Step 2**			
		Father encouragement × mother encouragement (within)	1.65 (–2.03 to 6.29)	2.56 (–1.66 to 8.35)
		Father encouragement × mother encouragement (between)	0.30 (–6.63 to 7.71)	–0.80 (–9.80 to 5.94)
**Random effect standard deviations**				
	**Step 1**			
		Intercept	2.98 (0.96 to 6.56)	1.81 (0.15 to 4.46)
		Father encouragement	2.52 (0.14 to 6.78)	1.14 (0.04 to 3.61)
		Mother encouragement	1.16 (0.04 to 3.75)	2.40 (0.15 to 6.62)

^a^F/V: fruits and vegetables.

**Table 3:** Multilevel model results (in log-odds) of parental support (preparing fruit and vegetables, taking the child to be physically active) predicting child outcomes of fruit and vegetables consumption and physical activity.

				Child F/V^a^ consumption (n=213)		Child physical activity (n=206)
**Fixed effects**						
	**Step 1**					
		Intercept	–3.58 (–7.84 to –0.69)		–2.57 (–5.81 to –0.26)	
		Father support (within)	1.57 (–0.86 to 4.66)		0.41 (–2.17 to 3.23)	
		Mother support (within)	0.85 (–1.14 to 3.38)		3.02 (–0.17 to 7.63)	
		Father support (between)	0.02 (–5.73 to 5.74)		1.17 (–4.28 to 7.82)	
		Mother support (between)	3.13 (–3.00 to 13.02)		–0.09 (–4.98 to 4.97)	
	**Step 2**					
		Father support × mother support (within)	3.29 (–0.77 to 9.53)		–1.28 (–6.19 to 3.10)	
		Father support × mother support (between)	0.44 (–7.33 to 9.08)		–0.04 (–7.81 to 7.66)	
**Random effect standard deviations**						
	**Step 1**					
		Intercept	3.49 (1.24 to 7.66)		2.56 (0.47 to 5.88)	
		Father support	1.46 (0.05 to 4.73)		1.55 (0.05 to 4.97)	
		Mother support	1.07 (0.04 to 3.48)		2.71 (0.13 to 7.97)	

^a^F/V: fruits and vegetables.

## Discussion

### Principal Findings

This exploratory study investigated the within-subject and between-subject independent and interactive effects of maternal and paternal encouragement and preparation of F/V and maternal and paternal encouragement of and taking their child to be physically active on their child’s self-reported F/V intake and physical activity engagement. There were no significant between-subject associations for (1) maternal or paternal encouragement of or preparation of F/V on children’s consumption or (2) maternal or paternal encouragement of or taking the child to be physically active on children’s physical activity. However, given the small number of participants, this study provides insufficient power for between-subject associations. Within-subjects results indicated that greater than usual maternal encouragement was associated with children’s greater likelihood of EMA-reported F/V consumption in the past 2 hours; however, paternal encouragement was not associated with children’s likelihood of reporting F/V consumption. Additionally, greater than usual maternal encouragement and greater than usual paternal encouragement were independently associated with greater likelihood of children’s EMA-reported physical activity in the past 2 hours. No within-subjects results for parental support (ie, taking the child someplace to be physically active, preparing F/V for the child) were significant.

Our previous EMA study using the same MATCH participants without fathers’ data showed the positive relationship of maternal encouragement with children’s subsequent F/V intake [11], but cross-sectional between-subjects research conducted among Norwegian families (85% mothers, children ages 10 to 11 years) indicated no relationship between parent-reported encouragement of F/V consumption and child-reported F/V intake [30]. In a similar between-subjects study conducted among Icelandic families (85% mothers, children aged 11 years), there was either no relationship between child-reported parental encouragement for F/V consumption and child-reported F/V intake [31] or a negative relationship between maternal-reported encouragement and children’s self-reported F/V intake [31]. In our study, mothers and fathers independently reported their encouragement of F/V consumption and children self-reported F/V intake, aligning with the parent-reported cross-sectional results and EMA results. Given the mixed findings in the literature, future research should engage more families and explore within- and between-effects from individual influences provided by mothers and fathers.

Another cross-sectional study that examined differences in children’s F/V consumption by race/ethnicity indicated greater child-reported social support for F/V intake among non-Hispanic White children compared to African American and Hispanic children [32]. The authors created a composite parental social support score that included several measures: providing children with prepared F/V, eating F/V, encouraging the child to eat F/V, and wanting the child to eat F/V. Stratified analyses showed that the greater the support provided, the higher reported vegetable consumption among Hispanic children [32]. In this study, we were not able to stratify our analyses by ethnicity due to the small sample size. However, nearly half of the children in our sample identified as Hispanic/Latino, illustrating that maternal encouragement of F/V consumption may be well received by both non-Hispanic White children and Hispanic/Latino children.

This study did not find statistically significant effects of mothers’ preparation or fathers’ preparation of F/V on children’s F/V intake. Previous longitudinal EMA research reported that greater than usual maternal preparation of F/V and greater maternal preparation of F/V when compared to other mothers resulted in increased odds of child-report of F/V consumption [11]. Results from other cross-sectional research indicated a positive association between adolescent-reported F/V consumption and fathers’ parenting practices that included preparation of F/V [33]. The lack of a relationship between F/V preparation and child F/V consumption may be due to the proximity of children’s ages to adolescence (age_mean_ 12.0 years, SD 0.7) in this study. As children age, they gain more independence for preparing their own snacks and thus, their parents may not spend as much time preparing F/V for them [34]. Additionally, the provision of F/V as snacks may not require extensive preparation, and thus parents may not view this as a considerable amount of time.

In this study, paternal encouragement of physical activity was associated with greater child-reported physical activity. Thus, fathers may play a larger role in supporting children’s physical activity than their intake of F/V. One study examined sports participation among low socioeconomic status youth and reported that strong paternal influence may be salient to encouraging children’s participation [35]. Previous qualitative research conducted among mothers indicates that they perceive fathers to play an active role in their children’s physical activity including encouraging the child to perform physical activity [36]. Additionally, in our study, maternal encouragement of physical activity was also associated with children’s physical activity. Our findings are also supported by previous longitudinal EMA research using the same MATCH participants (without fathers’ data) that indicates children’s MVPA levels were higher when mothers reported encouraging their child to be physically active within the same 2-hour window [12]. Our results also align with previous research indicating that maternal support for physical activity was positively associated with children’s device-measured activity levels [37]. In this study, parental encouragement plays a larger role for physical activity promotion rather than F/V intake. This may reflect differences in these behaviors such as the potential planning required to facilitate children’s physical activity, whereas home availability of F/V may not necessitate promotion. Thus, children may make more independent choices related to diet but still rely on parents for physical activity (eg, driving child somewhere to be physically active).

In contrast to parental encouragement, there were no statistically significant effects of the parent providing support by taking a child somewhere to be physically active on child-reported PA. Our previous EMA research using the same MATCH participants (without fathers’ data) indicates that children were more physically active when mothers reported taking their child somewhere to be physically active [12]. Cross-sectional research that examined the relationship between activity-related parental logistic support included a measure of taking the child somewhere to be physically active. Findings showed mothers had higher mean levels of support for girls’ physical activity compared to fathers, but there was no relationship among paternal and maternal logistical support and objectively measured child physical activity [18]. These contrasting findings may be due to the lack of separation of support into individual components in previous studies along with variability in reporting children’s physical activity [38,39]. For example, a study examining the cross-sectional associations between parenting practices and children’s pedometer-assessed physical activity combined measures for instrumental support (eg, taking the child somewhere to be physically active) and emotional support (eg, encouraging the child to be physically active). Results indicated that child-reported parent support was positively significantly associated with both boys’ and girls’ physical activity [38]. Another study found children’s perception of parental support was positively associated with both boys’ and girls’ questionnaire-assessed physical activity [39]. Future research should consider incorporating both child report of mothers’ and fathers’ support for physical activity and child perceptions of parental support to inform on children’s interpretations of parenting behaviors that may affect their own physical activity and identify areas on which to intervene (eg, just-in-time parenting interventions) at the family level.

### Strengths and Limitations

There has been limited research on the role of fathers’ parenting practices (ie, encouragement and support) on children’s energy balance behaviors. Thus, a major strength of this study includes the triadic design within everyday family contexts that allowed assessment of the independent and interactive influences of maternal and paternal parenting weight-related practices on children’s diet and physical activity. Additionally, the use of EMA methods to assess parenting and children’s behaviors in real time is an additional strength. However, this study is not without limitations. Due to the small sample size, we are unable to generalize our study findings to a broader population. Additionally, we are unable to stratify our results to assess potential moderators such as child sex or ethnicity. The 2-hour time window used for analysis may not have perfectly overlapped among mother-, father-, and child-reported behaviors. We also cannot establish temporality within the 2-hour period. For example, it is unknown whether the mother and/or father encouraged the child to be physically active or eat F/V before or after the child reported engaging in physical activity or eating F/V. We also did not control for environmental contexts including weather conditions, physical location, and level/type of physical activity, which have been shown to influence activity levels [40]. Mothers and fathers were not provided with a specific definition for the term *encourage* as it pertained to children’s physical activity and healthy eating. Thus, answering questions related to encouragement of consumption of F/V and physical activity may have been interpreted in various ways. However, they were given the opportunity to ask clarifying questions during orientation to the study and provided with a contact number to ask any questions that arose. We are unaware whether the mother or father ate or exercised in the presence of their child. It is possible that the child reported eating or being physically active because these behaviors were not only encouraged but also modeled by the parent to their child. Last, parenting practices and support, along with children’s outcomes of F/V consumption and physical activity, were self-reported rather than objectively measured and therefore open to recall bias and social desirability bias [41].

### Conclusion

Our results indicate children who are encouraged to eat F/V report greater F/V consumption and children who are encouraged to be physically active report greater physical activity. Furthermore, fathers’ encouragement of physical activity has an independent effect to mothers’ encouragement of physical activity on children’s EMA-reported physical activity. These findings have implications for future just-in-time parenting interventions to promote children’s F/V consumption and physical activity. For example, prompting of parental encouragement from both mothers and fathers during times when children can eat F/V and engage in physical activity may result in greater consumption of F/V and higher levels of physical activity.

## Abbreviations

EMA: ecological momentary assessment
F/V: fruit and vegetable
MATCH: Mothers and Their Children’s Health Study
MVPA: moderate-to-vigorous physical activity
USC: University of Southern California
